# FKBP10 Promotes the Muscle Invasion of Bladder Cancer via Lamin A Dysregulation

**DOI:** 10.7150/ijbs.105265

**Published:** 2025-01-01

**Authors:** Xupeng Zhao, Jichen Wang, Shuo Tian, Lu Tang, Shouqing Cao, Jiali Ye, Tianwei Cai, Yundong Xuan, Xu Zhang, Xiubin Li, Hongzhao Li

**Affiliations:** 1 School of Medicine, Nankai University, Tianjin, China.; 2 Department of Urology, Chinese PLA General Hospital, Beijing, China.; 3 Medical School of Chinese PLA, Beijing, China.

**Keywords:** FKBP10, prelamin A, lamin A, nuclear atypia, muscle-invasive bladder cancer

## Abstract

Bladder cancer (BC) is a prevalent urinary malignancy and muscle-invasive bladder cancer (MIBC) is particularly aggressive and associated with poor prognosis. One of MIBC features is the nuclear atypia. However, the molecular mechanism underlying MIBC remains unclear. Here, we find that FKBP10 is significantly upregulated in MIBC tissues and correlated with metastasis and poor outcomes. FKBP10 promotes tumor cell invasion, migration, and metastasis, but not proliferation. Notably, FKBP10 enhances the nuclear atypia of BC cells. Mechanistically, FKBP10 interacts with prelamin A and hinder the nuclear entry of prelamin A, thereby leading to the decrease in the nuclear lamin A, a key factor involved in nuclear atypia. In human BC tissues, nuclear lamin A is downregulated and negatively correlated with FKBP10 expression. Overall, our findings demonstrate that the FKBP10/prelamin A/lamin A axis contributes to MIBC.

## Introduction

Bladder cancer (BC) is a common malignancy of the urinary system and ranks ninth in terms of the global cancer incidence 1. Approximately 25-30% of the patients with BC are originally diagnosed with muscle-invasive bladder cancer (MIBC) 2. MIBC is associated with lymph node and organ metastases, leading to poor prognosis 3. It is characterized by heightened nuclear atypia, which serves as a diagnostic hallmark 4. However, the mechanism underlying the nuclear atypia in BC remains unclear.

A key factor involved in nuclear atypia is nuclear envelope rigidity. The nuclear envelope separates the cytoplasm from the nucleus and consists of the inner nuclear membrane, outer nuclear membrane, nuclear pores, and nuclear lamina 5. The nuclear lamina, which is primarily composed of A-type lamins (lamins A and C) and B-type lamins (lamins B1 and B2), is crucial for maintaining nuclear rigidity and morphology 6. Lamin A deficiency leads to reduced nuclear rigidity, increased deformability and a higher incidence of malformed nuclei 7. This increased nuclear deformability facilitates the migration of tumor cells through narrow spaces, enhancing their invasive potential 8. Indeed, previous studies have demonstrated that lamin A downregulation results in the invasion and metastasis of various cancers, such as Ewing sarcoma, breast cancer and ovarian cancer 9-12. Despite these findings, however, the regulatory mechanism underlying the downregulation of lamin A in BC remains unclear, particularly in the context of nuclear atypia.

FK506 binding protein 10 (FKBP10) is a member of the FKBP family with peptidyl-prolyl cis-trans isomerase activity 13. FKBP10 resides in the endoplasmic reticulum, where it assists in protein folding 14. Recent studies have shown the upregulation of FKBP10 in various cancers, including clear cell renal cell carcinoma, colorectal cancer, gastric cancer and lung adenocarcinoma 15-18. FKBP10 can promote cancer cell proliferation, migration, invasion and metastasis 19-22. However, dysregulation of FKBP10 in BC has not been explored.

In this study, we investigated the mechanisms underlying the nuclear atypia in BC. First, we identified that FKBP10 was significantly upregulated in MIBC compared to non-muscle-invasive bladder cancer (NMIBC). Next, we found that although FKBP10 did not promote cancer cell proliferation, FKBP10 promoted the migration and invasion of cancer cells, as well as the infiltration into the muscle layer in orthotopic bladder tumor model. Third, we screened the proteins interacting with FKBP10 via mass spectrometry, and identified that FKBP10 can directly bind to prelamin A in the endoplasmic reticulum (ER). Finally, we showed that FKBP10 overexpression resulted in the retention of more prelamin A in the ER, thus reducing its level in the nucleus. Consequently, the reduction of the nuclear prelamin A led to the decease of lamin A, which is responsible for nuclear integrity.

## Methods

### Patients and tissue samples

A total of 100 BC samples and paired with adjacent normal tissue samples were selected from January 2015 to December 2017 to create TMAs. 8 pairs of fresh BC and adjacent normal bladder tissues were collected and promptly frozen in liquid nitrogen for protein extraction. Informed consent was obtained from all patients and the study was approved by the Institutional Review Board of PLA General Hospital and have been performed in accordance with the ethical standards as laid down in the 1964 Declaration of Helsinki and its later amendments. Diagnosis of all specimens was independently confirmed by three experienced pathologists.

### Cell cultures

HEK293T cells and human BC cell lines UMUC3 and T24 sourced from National Platform of Experimental Cell Resources for Sci-Tech (Beijing, China). HEK293T and UMUC3 cells were maintained in DMEM (Procell) medium with 10% fetal bovine serum (FBS)(Procell), while T24 cells were cultured in RPMI 1640 (Procell) medium with 10% FBS. All cells were incubated at 37°C with 5% CO_2_ and confirmed to be free of mycoplasma contamination.

### Lentiviral-infected stable cell lines

Short hairpin RNAs (shRNAs) targeting FKBP10 were designed and inserted into the PLKO.1 vector as double-stranded oligonucleotides. The sequences are listed in Supplement Table 1. Lentiviruses were produced using HEK293T cells following the manufacturer's instructions (Polyplus). The lentiviral particles collected at 48 h and 72 h, then added to the target cells with polybrene (2μg/ml, Solarbio). Subsequently, puromycin (2μg/ml, Solarbio) was added for a 72-hour screening period to establish stable cells.

### Plasmids and transient transfection

The construction of overexpression plasmids included FKBP10-Flag (wild-type and truncated-type), FKBP10-6*His (wild-type), and prelamin A-HA (wild-type and truncated-type). The cDNA from HEK293T cells was utilized as a template for PCR using high-fidelity PCR enzymes (Vazyme). The PCR product was verified via Sanger sequencing and then cloned into the PLVX-Puro vector. Transient transfection was performed using jetPRIME (Polyplus) in UMUC3 or HEK293T cells. Specifically, when the cell density of UMUC3 or HEK293T reached 70%, the transfection reagent and plasmid were mixed at a 2:1 ratio for transfection.

### siRNA transfection

siRNAs targeting LMNA were purchased from Genepharma and transfected into UMUC3 cells using jetPRIME (Polyplus) following the provided instructions. The specific siRNA sequence can be found in Supplementary Table 1.

### Western blot analysis

Total protein extracts were prepared using RIPA buffer (Solarbio) supplemented with protease and phosphatase inhibitors. The cytoplasmic and nuclear separation test was carried out as per the kit instructions (Abbkine, #KTP3001). Cells used in cytoplasmic and nuclear separation tests were treated with 5 μM lonafarnib (MCE, #HY-15136) for 48 hours in advance. Protein concentrations were determined using the BCA assay (Solarbio). Equal amounts of protein (20 μg) were separated by SDS-PAGE and transferred onto PVDF membranes. Membranes were blocked with 5% non-fat milk in TBST and incubated overnight at 4°C with primary antibodies against FKBP10 (1:8000, Proteintech, #12172-1-AP), prelamin A (1:1000, Merck, #MABT858), lamin A/C (1:30000, Proteintech, #10298-1-AP), Histone H3 (1:8000, Proteintech, #17168-1-AP), DYKDDDK (1:20000, Proteintech, #66008-4-Ig), HA (1:20000, Proteintech, #81290-1-RR), 6*His (1:10000, Proteintech, #66005-1-Ig) and GAPDH (1:20000, Proteintech, #81640-5-RR). After washing, membranes were incubated with HRP-conjugated secondary antibodies and developed using an ECL system.

### Immunohistochemistry (IHC)

Tumor tissue sections (5 μm) were deparaffinized, rehydrated, and subjected to antigen retrieval using citrate buffer. Endogenous peroxidase activity was inhibited with 3% H_2_O_2_. The sections were then incubated overnight at 4°C with primary antibodies against FKBP10 (Proteintech, 1:200, #12172-1-AP) and lamin A/C (1:500, Proteintech, #10298-1-AP), followed by incubation with a horseradish peroxidase-conjugated secondary antibody. Visualization was achieved using DAB (Maixin Biotech) substrate, and nuclei were counterstained with hematoxylin. IHC scoring was conducted by three pathologists to assess the percentage of positive cells and staining intensity. The proportion score was categorized based on the proportion of positive cells as follows: 0 (< 10%), 1 (10-25%), 2 (26-50%), 3 (51-75%), and 4 (>75%). Staining intensity was scored as: 0 (no staining), 1 (light brown), 2 (brown), and 3 (dark brown). H-score= (percentage of cells of proportion score 1×1) + (percentage of cells of proportion score 2×2) + (percentage of cells of proportion score 3×3), the maximum H-score is 300.

### Hematoxylin/eosin (HE) staining

Following deparaffinization and rehydration, the tissue sections (5 μm) were stained with hematoxylin for 5 minutes, differentiated with a hydrochloric acid-alcohol solution for 1 second, stained with eosin for 90 seconds, dehydrated, cleared, and sealed.

### Immunofluorescence (IF)

Cell climbings and frozen slices were fixed with 4% formaldehyde, permeabilized with 0.5% Triton X-100, and blocked with 5% Goat serum blocking solution. Cell climbings were then incubated with primary antibodies GFP (CST, 1:200, ab213511), mCherry (abcam, 1:100, ab213511), FKBP10 (Proteintech, 1:400, #12172-1-AP) and prelamin A (1:1000, Merck, #MABT858), followed by Alexa Fluor-conjugated secondary antibodies (488-conjugated Goat anti-Mouse IgG and 594-conjugated Goat anti-Rabbit IgG (1:500, ABclonal). Nuclei were stained with DAPI (Abcam, ab104139), and images were captured using High Intelligent and Sensitve SIM.

For paraffin-embedded sections, the samples were deparaffinized, rehydrated, and subjected to antigen retrieval using citrate buffer. Endogenous peroxidase activity was inhibited with 3% H₂O₂. Following a 30-minute blocking step with goat serum at room temperature, the sections were incubated overnight at 4°C with primary antibodies against FKBP10 (Proteintech,1:400, #12172-1-AP) or prelamin A (1:1000, Merck, #MABT858), incubation with a horseradish peroxidase-conjugated secondary antibody. Visualization was performed using TSA dye in the 488 channel for 5 minutes. After confirming the signal under a fluorescence microscope, the sections were treated with elution buffer at 37°C for 20 minutes. The sections were then blocked again with goat serum at room temperature for 30 minutes and incubated overnight at 4°C with a primary antibody against GRP78 (Proteintech,1:200, #11587-1-AP). The procedure concluded with incubation with an HRP-conjugated secondary antibody, followed by visualization using TSA dye in the 594 channel for 3 minutes.

### Wound healing assay

BC cells were grown to confluence in 6-well plates and a linear wound was created using a 200μl pipette tip. Images were captured at 0 and 24 hours post-wounding using a phase-contrast microscope. Wound closure was quantified by measuring the remaining open wound area using ImageJ software.

### CCK8 assay

1×10^3^ BC cells were inoculated into each well of a 96-well plate. CCK8 reagent (Abbkine, #KTA1020) was added to each well at 0, 24, 48, and 72 hours, followed by incubation at 37 ℃ for 2 hours prior to measurement absorbance at 450 nm.

### Transwell assay

For migration assays, cells (1 × 10^4^) were seeded in the upper chamber of Transwell inserts with 3μm ,5μm and 8μm pores in medium with 10% FBS. For invasion assays, the upper chamber was coated with Matrigel. After 24 hours, cells on the upper surface were removed, and those that had migrated to the lower surface were fixed, stained with crystal violet, and counted.

### Protein-protein docking

We searched FKBP10 and prelamin A in the Uniprot database (https://www.uniprot.org/). The species are both human and the Uniprot IDs are Q96AY3 and Q9UHQ1. As there are no resolved full-length crystal structures available for these proteins, their three-dimensional structures were predicted using alphafold2, followed by energy minimization using the OPLS4 force field. Molecular docking was performed using the standard mode of the Protein-Protein docking (Piper) protein-protein docking module in Schrödinger app.

### Immunoprecipitation (IP) and Mass Spectrometry (MS) analysis

For IP experiments, T24 and UMUC3 cells were lysed using NP-40 lysis buffer (Solarbio) with Cocktail protease Inhibitor (Selleck, # B14001) to extract whole cell lysates. The lysate was then treated with FKBP10 antibody (Proteintech, #12172-1-AP) or a negative control IgG (MCE, HY-P73904) at 4°C overnight, followed by incubation with protein A/G magnetic beads (MCE, HY-K0202) for 4 hours at 4°C. After extensive washing with PBST solution (1×PBS, 0.5% Tween-20, pH 7.4), elution was performed by heating with 1×SDS-PAGE loading buffer for subsequent mass spectrometry analysis.

MS analysis was conducted by Beijing Qinglian Biao Company using a quadrupole Orbitrap mass spectrometer (Orbitrap Exploris™ 480, Thermo Fisher Scientific, Bremen, Germany). MS data were analyzed using the Proteome Discoverer suite (version 2.4, Thermo Fisher Scientific). with the UniProtKB human proteome database.

### Co-Immunoprecipitation (Co-IP)

Following transfection of the target plasmid into HEK293T cells after 48h, the cells were lysed using NP-40 lysis buffer (Solarbio) containing Cocktail protease Inhibitor (Selleck, # B14001) to extract total protein. The lysate was then incubated with anti-Flag magnetic beads (Selleck, #B26101), anti-HA magnetic beads (MCE, HY-K0201), or protein A/G magnetic beads (MCE, HY-K0202) coated with 6*His (Proteintech, #66005-1-Ig) overnight at 4°C. Subsequently, the beads were washed thoroughly with PBST before adding 1×SDS-PAGE loading buffer. The elution was performed by heating at 95°C for western blot analysis.

### Pull-down assay

The 6*His-tagged FKBP10 protein and the HA-tagged prelamin A protein were overexpressed and purified in HEK293T cells. Protein A/G magnetic beads (MCE, HY-K0202) coated with 6*His (Proteintech, #66005-1-Ig) were used to capture the FKBP10 protein, followed by incubation at 4°C overnight. Subsequently, the purified prelamin A protein was added and further incubated at 4°C for 4 hours. Elution for western blot analysis was performed using 1×SDS-PAGE loading buffer.

### Animal experiments

All animal experiments were complied with the ARRIVE guidelines and approved by the Ethics Committee of PLA General Hospital.

In the orthotopic bladder tumor model, 1×10^6^ cells suspended in 50 µl of PBS were injected into the bladder of 6-week male nude mice. The 34G syringe was inserted into the right lateral bladder wall, and the left wall was scraped 8-10 times before withdrawal. After a period of 3 weeks, the mice were anesthetized, sacrificed, and their bladders were harvested and weighed.

In the competitive growth experiment, 5×10^5^ scramble UMUC3 cells labeled with mCherry and 5×10^5^ shFKBP10 UMUC3 cells labeled with GFP were mixed and suspended in 50 µl of PBS. The procedure was otherwise identical to that of the orthotopic bladder tumor model.

For the xenograft tumor model, 1×10^6^ cells were subcutaneously injected into either 6-week male nude mice. Following 4 weeks, the mice were anesthetized, sacrificed and the tumor tissues were isolated and weighed.

To establish a lung metastasis model, 1×10^6^ cells suspended in 100 µl of PBS were injected into the tail vein of the 6-week-old male nude mice. After 8 weeks, the mice were anesthetized and euthanized. The lungs were then isolated, weighed, and photographed. All specimens underwent formalin fixation and paraffin embedding for subsequent IHC and HE staining experiments.

### Bioinformatic analysis

The proteome data of BC in the Chinese population was sourced from published literature. Data are grouped according to tumor and normal bladder tissue, NMIBC and MIBC. The data was normalized using the normalize Between Arrays function of the limma package, and differential analysis was conducted to identify gene intersections. Subsequently, the GO SemSim [2.22.0] function was employed to calculate the semantic similarity between significant biological processes.

### Statistical analysis

Data are presented as mean ± SD from at least three independent experiments. Statistical significance was determined using a two-tailed unpaired t-test or one-way ANOVA with Tukey's post-hoc test using Prism software (GraphPad). A P-value of less than 0.05 was considered statistically significant.

## Results

### FKBP10 is associated with muscle invasion and poor prognosis in BC

**Figure 1:**
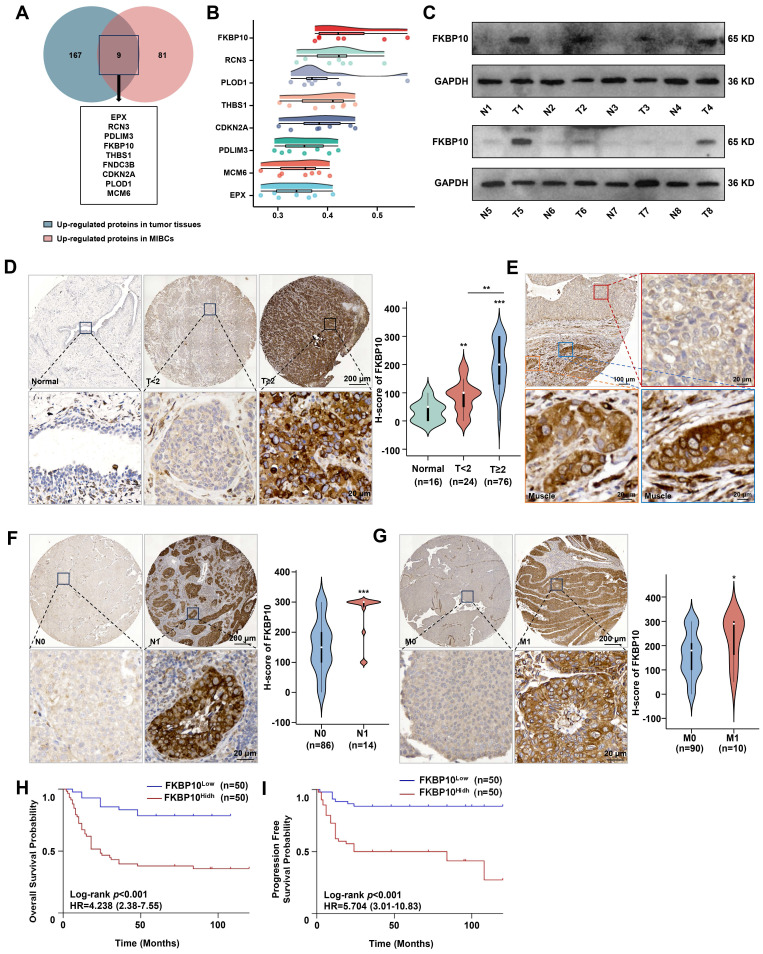
** FKBP10 is highly expressed in MIBC. A.** Venn-diagram analysis of genes from BC proteomic data in Chinese population. **B.** Gene similarity analysis based on GO SemSim. **C.** Western blot detection of FKBP10 protein expression in normal bladder tissues and BC tissues. Tissues 1-6 and Tissue 8 are MIBC, tissue 7 is NMIBC.** D.** IHC analysis of FKBP10 expression in BC tissues of different T stages. **E.** Representative fields of both mucosal tumors and muscle-invasive tumors. The red box represents mucosal bladder tumors, and the blue and orange boxes represent muscle-invasive tumors. **F, G.** IHC analysis of FKBP10 expression in BC tissues of different N stages **(F)** and M stages **(G)**. **H, I.** Kaplan-Meier curve of OS **(H)** or PFS **(I)** between FKBP10 high expression group and low expression group. Data are expressed as mean ± standard deviation. Statistical analysis was performed using unpaired two-tailed Student's t test. * *p*<0.05, ** p<0.01, *** *p*<0.001.

Because proteins, rather than RNA, play a significant role in tumor progression, our study focused on identifying proteins that are upregulated in tumor tissues, particularly those that show increased expression in MIBC compared to NMIBC. By analyzing the proteomic data from 145 human BC tissues 23, we identified 9 proteins that were significantly upregulated in MIBC compared with NMIBC** (Fig. 1A)**. Among them, FKBP10 was the top protein associated with tumor progression **(Fig. 1B)**. This suggests that FKBP10 may be a key molecule involved in the muscle invasion in BC. Next, we found that FKBP10 upregulation was correlated with poor prognosis **(Fig. S1A)**. To confirm this finding, we used another 8 BC and adjacent tissues to detect the protein level of FKBP10 by western blot. Consistently, we found the FKBP10 upregulation in most MIBC tissues** (Fig. 1C)**. Furthermore, IHC from 100 BC samples verified the upregulation of FKBP10 in MIBC compared with NMIBC** (Fig. 1D)**. Intriguingly, cancer cells displayed the elevated FKBP10 expression when invading into the muscle layer of the bladder **(Fig. 1E)**. In addition, FKBP10 overexpression was associated with lymph node** (Fig. 1F)** and distant metastasis **(Fig. 1G)**. As expected, the FKBP10 protein levels were negatively correlated with overall survival (OS) **(Fig. 1H)** and progression-free survival (PFS) **(Fig. 1I)**. These findings indicated that FKBP10 is associated with muscle invasion and poor prognosis in BC, and may be involved in MIBC.

### FKBP10 promotes the migration and invasion of BC cells

**Figure 2:**
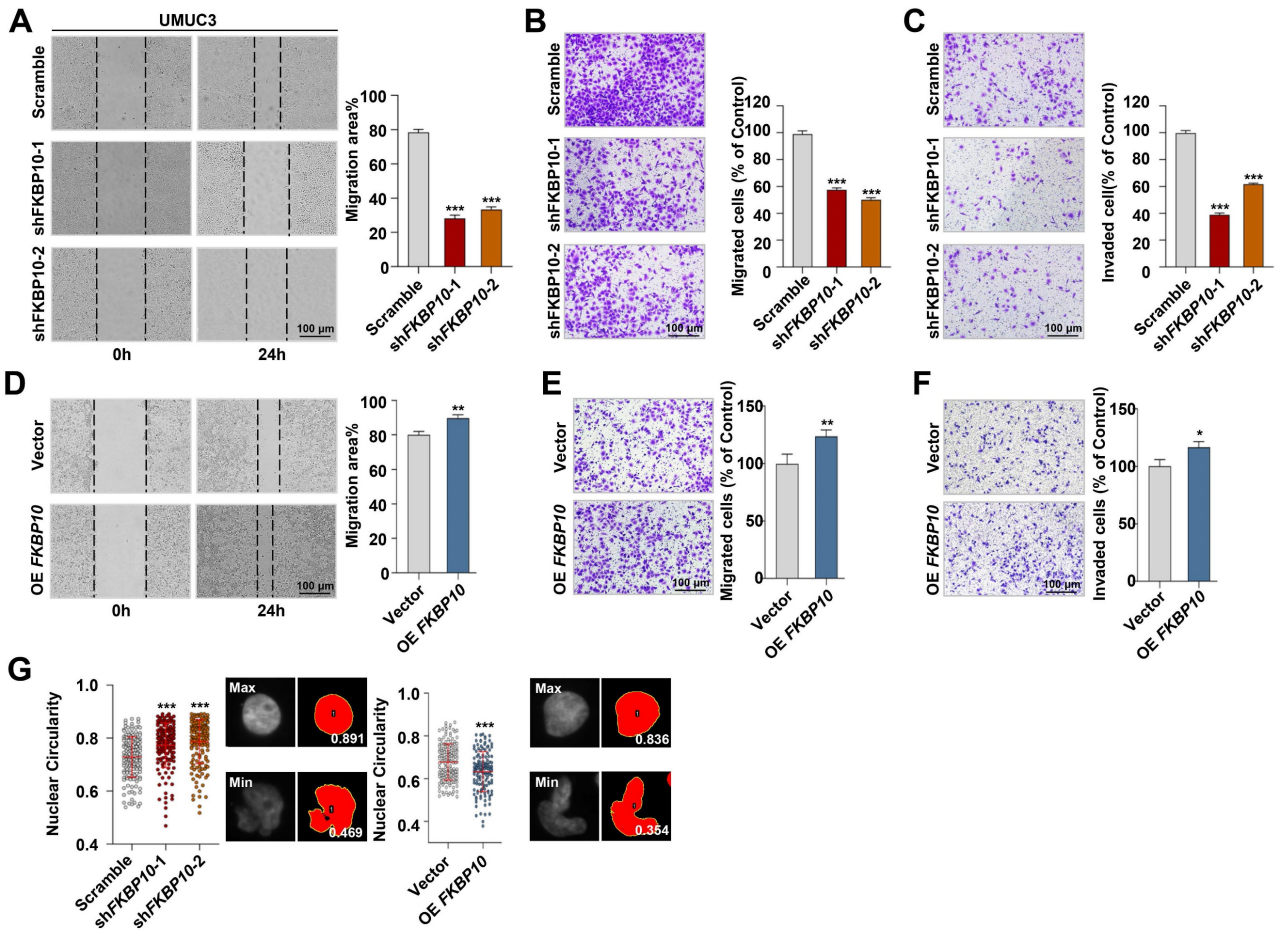
** FKBP10 promotes invasion and migration of BC *in vitro*. A.** Wound healing assay of scramble *vs.* shFKBP10 UMUC3 cells (n=3).** B, C.** Transwell migration and invasion assays of scramble *vs.* shFKBP10 UMUC3 cells (n=3). **D-F.** Wound healing **(D)** and Transwell assays **(E, F)** of vector *vs.* FKBP10-overexpressing UMUC3 cells (n=3). **G.** UMUC3 cells with FKBP10 knocked-down or overexpression were stained with DAPI, and the nuclear circularity of each nucleus was calculated using Image J software. The red bar represents the mean and standard deviation (left). Representative pictures of nuclei with the largest and smallest circularity scores are shown(right). Data are expressed as mean ± standard deviation. Statistical analysis was performed using unpaired two-tailed Student's t test. * p<0.05, ** *p*<0.01, *** p<0.001.

To explore the biological functions of FKBP10 in BC, we established stable cell lines with either FKBP10 knockdown or overexpression. This efficiency was confirmed by western blot **(Fig. S2A)**. First, we found that FKBP10 expression did not affect the proliferation of T24 or UMUC3 cells **(Fig. S2B)**. However, wound healing and transwell assays indicated that the knockdown of FKBP10 significantly inhibited the migration and invasion of UMUC3 **(Fig. 2A-C)** and T24 cells **(Fig. S2C-E)**. Conversely, FKBP10 overexpression promoted the migration and invasion of UMUC3 cells **(Fig. 2D-F)**. Notably, we revealed that FKBP10 overexpression promoted nuclear atypia, whereas FKBP10 knockdown inhibited the nuclear atypia in UMUC3 cells **(Fig. 2G)**.

**Figure 3:**
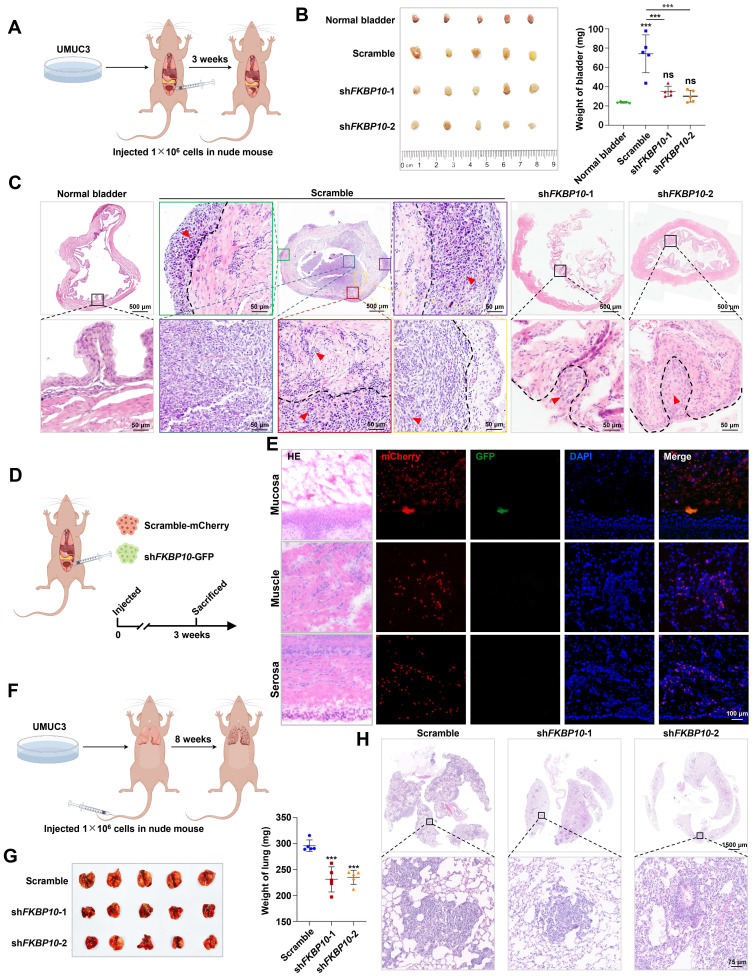
** FKBP10 promotes invasion and migration of BC *in vivo*. A.** Schematic of the orthotopic bladder tumor model in nude mice. **B.** Gross view of the bladders and statistical analysis of bladder weight (n=5). **C.** Representative pictures of HE staining of orthotopic tumors in nude mice bladders. In scramble group, blue boxes marked mucosal tumors, yellow boxes mark submucosal invasive tumors, red, green and purple boxes marked muscle invasive tumors. The black dotted line is the dividing line between different layers of the bladder, and the red arrow points to the tumor tissues. **D.** Schematic of the competitive growth model in nude mice.** E.** IF staining results of frozen bladder tissue sections. **F.** Schematic of the lung metastasis model. **G.** Gross view of the lungs and lung weight analysis (n=5). **H.** HE staining of lung metastatic lesions. Statistical analysis was performed using unpaired two-tailed Student's t test. * *p*<0.05, ** *p*<0.01, *** *p*<0.001.

Next, we used the subcutaneous xenograft model, the orthotopic bladder tumor model and the lung metastasis model to investigate the effect of FKBP10 on BC progression *in vivo*. In the subcutaneous xenograft tumor model, the tumor weight between the shFKBP10 and scramble groups was comparable **(Fig. S2F)**, which is consistent with the results of the proliferation assay **(Fig. S2B)**. However, in the orthotopic bladder tumor model, FKBP10 knockdown significantly reduced the overall bladder weight **(Fig. 3A, B)**. HE staining revealed that in the control group, cancer cells extensively invaded into muscle layer, whereas FKBP10-knockdown cells retained in the mucosal or submucosal layer **(Fig. 3C)**. We also conducted a competitive growth experiment using a mixture with the comparable control and knockdown cells, and found that the control BC cells but not FKBP10-knockdown cells were present in the bladder **(Fig. 3D, E)**, indicating that FKBP10 can promote the colonization and infiltration of BC cells. Finally, the FKBP10 knockdown group showed a significant reduction in the number and size of lung metastatic lesions** (Fig. 3F-H)**.

Taken together, these results suggested that FKBP10 plays a pivotal role in promoting the muscle invasion and metastasis of BC cells.

### FKBP10 interacts with prelamin A

**Figure 4:**
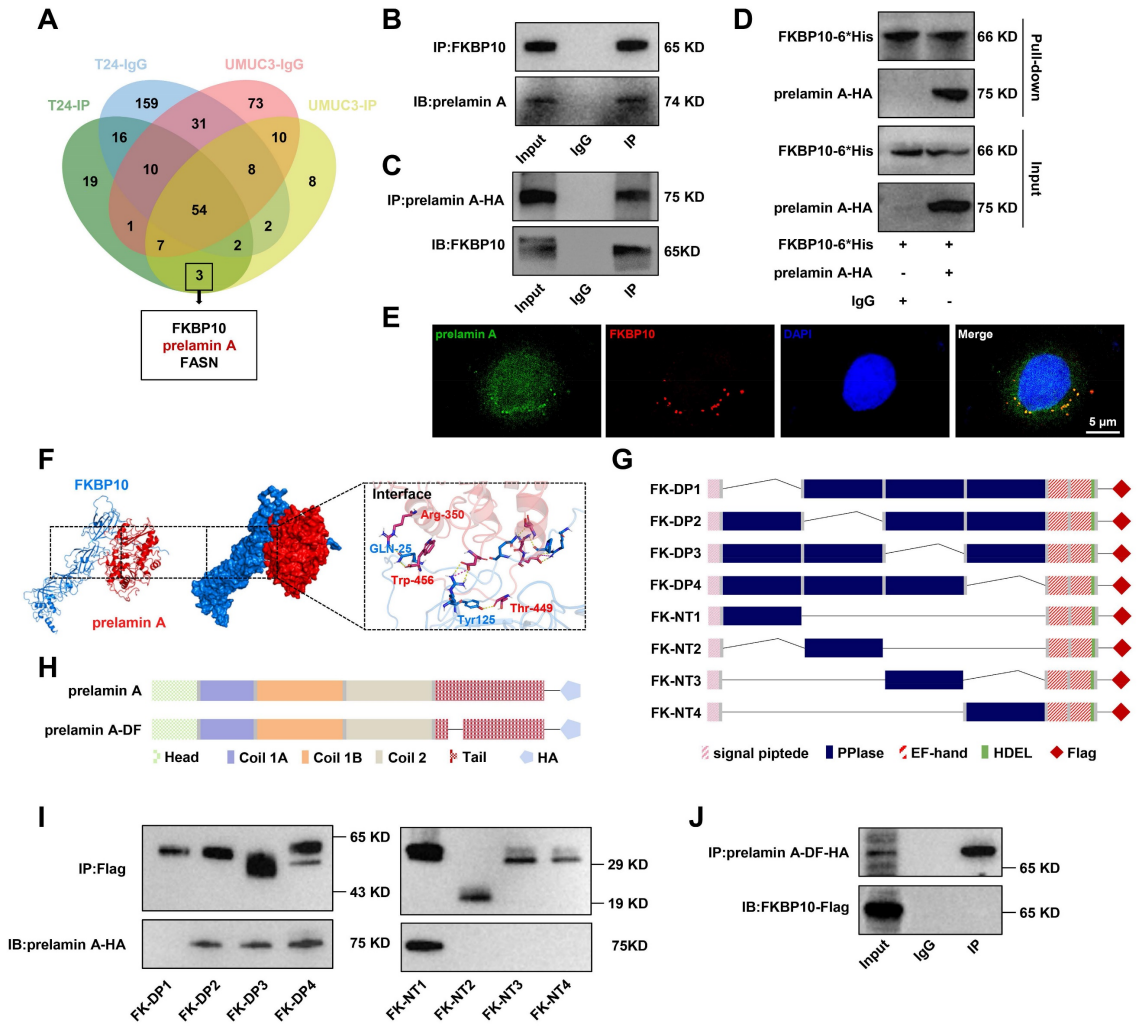
** FKBP10 directly binds with prelamin A. A.** Venn diagram of FKBP10-binding proteins from MS analysis. **B, C.** Co-IP analysis of FKBP10 and prelamin A interaction. **D.** *In vitro* pull-down confirmed that FKBP10 directly bound with prelamin A interaction. **E.** IF staining showed the binding of endogenous FKBP10 and prelamin A in UMUC3 cells. **F.** Virtual molecular docking of FKBP10 and prelamin A. **G, H** Schematic diagram of FKBP10 and prelamin A truncations**. I.** Co-IP experiments of exogenous prelamin A -HA with different FKBP10 truncations in HEK293T cells. **J.** Co-IP assays of the interaction between prelamin A-DF-HA and FKBP10-Flag in HEK293T cells.

To screen the downstream effectors of FKBP10, we performed IP followed by mass spectrometry in UMUC3 and T24 cells and identified a potential interaction between FKBP10 and FASN or prelamin A **(Fig. 4A)**. Subsequent Co-IP verified the interaction between FKBP10 and prelamin A **(Fig. 4B, C)**, but not FASN **(Fig. S3A)**.

As a precursor of lamin A, prelamin A is crucial for nuclear structure and integrity. Considering the function of FKBP10 in nuclear atypia, we focused on prelamin A. Co-IP assays showed that endogenous FKBP10 was able to bind with prelamin A **(Fig. 4B)**, but not lamin A/C **(Fig. S3B)**. Because of the lack of commercial antibodies against prelamin A for IP, we expressed HA-tagged prelamin A in UMUC3 cells. Co-IP experiments demonstrated an interaction between FKBP10 and prelamin A-HA **(Fig. 4C)**. Previous studies have shown that FKBP10 is located in the ER in lung cancer cells 24. Also, we found the specific location of FKBP10 in the ER of BC cells **(Fig. S4A)**. We further observed prelamin A present in ER of BC cells **(Fig. S4B)**. Importantly, the co-localization of FKBP10 and prelamin A was observed in BC cells **(Fig. 4E)**. Finally, pull-down experiment confirmed a direct interaction between FKBP10 and prelamin A **(Fig. 4D)**.

Next, we used AlphaFold to predict the potential interaction domains between the first PPIase domain of FKBP10 and the 350-450 amino acids (aa) of prelamin A **(Fig. 4F)**. We expressed different truncations of FKBP10-Flag and prelamin A-HA **(Fig. 4G, H)** in HEK293T cells. Co-IP verified that the first PPIase domain of FKBP10 and 350-450 aa of prelamin A was independent for their interaction **(Fig. 4I, J; Fig. S3C, D)**.

### FKBP10 sequesters prelamin A in the ER and inhibits its nuclear translocation

**Figure 5:**
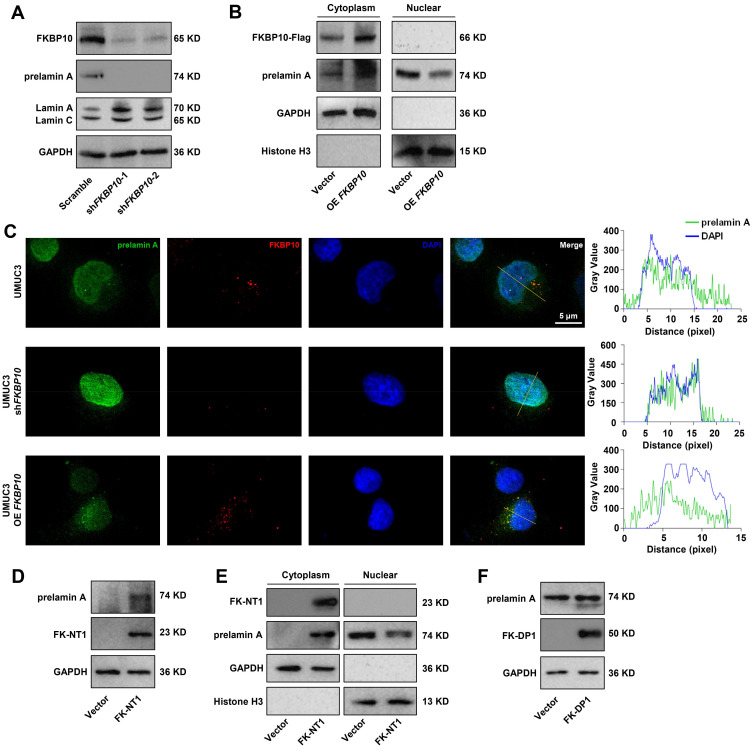
** FKBP10 inhibits prelamin nuclear translocation. A.** Western blot analysis of prelamin A and lamin A/C protein levels in scramble and shFKBP10 cells. **B.** Western blot analysis of cytoplasmic and nuclear fractions from scramble and shFKBP10 UMUC3 cells. Nuclear fractionation was validated using histone H3, and cytoplasmic fractionation was confirmed with GAPDH. **C.** IF analysis of prelamin A nuclear entry in FKBP10 knockdown/overexpressing cells. **D.** Western blot analysis of prelamin A protein levels in FKBP10-knockdown UMUC3 cells with or without FK-NT1 overexpression.** E.** Western blot analysis of cytoplasmic and nuclear fractions from FKBP10-knockdown UMUC3 cells with or without FK-NT1 overexpression. Nuclear fractionation was confirmed by detection of histone H3 and cytoplasmic fractionation by GAPDH. **F.** Western blot analysis of prelamin A protein levels in FKBP10-knockdown UMUC3 cells with or without FK-DP1 overexpression.

To investigate the effects of the interaction between FKBP10 and prelamin A, we assessed the expression level of prelamin A in UMUC3 cells. Western blot analysis revealed that FKBP10 knockdown led to the decrease in prelamin A. However, the lamin A expression increased** (Fig. 5A)**. Since lamin A is specifically located in the nucleus, the increase in lamin A expression was due to the increase in its precursor prelamin A that entered into the nucleus. Indeed, we found that FKBP10 overexpression resulted in the increase in prelamin A in the cytoplasm and a concomitant reduction in the nucleus** (Fig. 5B)**, suggesting that FKBP10 hindered the nuclear translocation of prelamin A. Supporting this finding, IF assays showed that FKBP10 knockdown reduced the cytoplasmic prelamin A and consequently elevated its nuclear level, and conversely, FKBP10 overexpression led to the increase in the cytoplasmic prelamin A and the decrease of the nuclear prelamin A **(Fig. 5C)**.

To further validate these findings, we transfected FKBP10-knockdown UMUC3 cells with two truncations of FKBP10, FK-NT1 and FK-DP1. The overexpression of FK-NT1, which retains the prelamin A-binding domain, resulted in the increase in cytoplasmic prelamin A and the decrease in its nuclear levels **(Fig. 5D, E)**. In contrast, overexpression of FK-DP1, which lacks prelamin A-binding domain, did not significantly affect the prelamin A level **(Fig. 5F)**. These results indicated that FKBP10 sequestered prelamin A in cytoplasm, thus preventing prelamin A from entering into nucleus and reducing nuclear lamin A levels.

### FKBP10 downregulates lamin A to promote the nuclear atypia, invasion, and migration of BC cells

**Figure 6:**
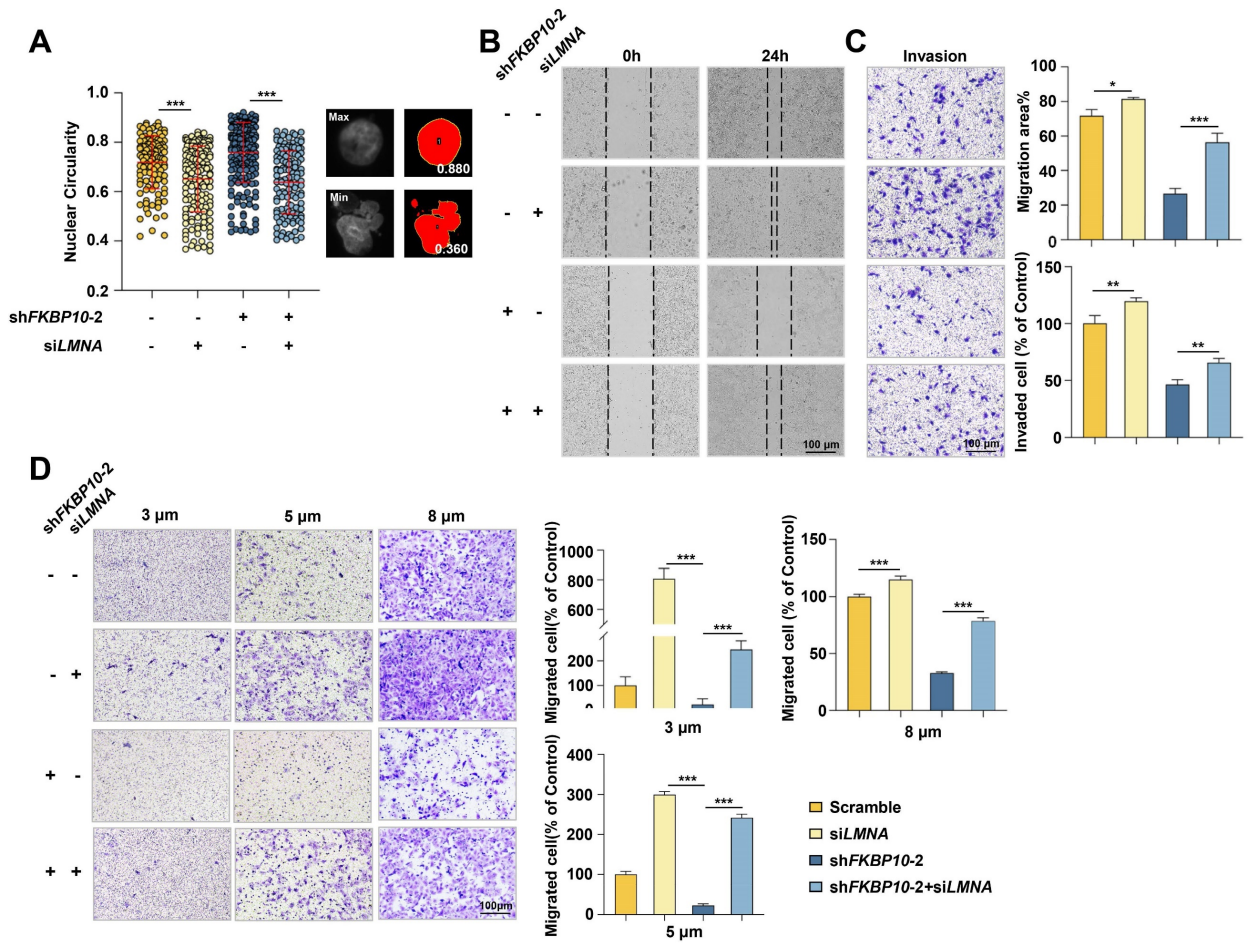
** Lamin A downregulation leads to nuclear atypia and promotes BC cells migration/invasion. A.** Image J analysis after DAPI staining to evaluate rescue of nuclear atypia in shFKBP10 UMUC3 cells transfected with LMNA siRNA. The red bar represents the mean and standard deviation (left). Representative pictures of nuclei with the largest or smallest circularity scores are shown(right).** B.** Wound healing experiments to assess rescue of migration ability in shFKBP10 UMUC3 cells by LMNA siRNA transfection (n=3). **C.** Transwell experiments to assess rescue of invasion ability in shFKBP10 UMUC3 cells by LMNA siRNA transfection (n=3). **D.** Representative pictures (left) and statistical results (right) of cell migration through chambers with different pore sizes in the Transwell experiment of shFKBP10 UMUC3 cells with LMNA siRNA through different pore sizes (n=3). Data are expressed as mean ± standard deviation. Statistical analysis was performed using unpaired two-tailed Student's t test. * *p*<0.05, ** p<0.01, ***.

Next, we investigated whether lamin A mediated the effect of FKBP10 on BC progression. FKBP10-knockdown UMUC3 cells were simultaneously transfected with LMNA siRNA. IF experiments demonstrated that lamin A knockdown led to nuclear atypia and LMNA knockdown partially inhibited the reduction of nuclear atypia caused by FKBP10 knockdown in UMUC3 cells **(Fig. 6A)**. Previous studies have demonstrated that lamin A deficiency enhances nuclear deformability, thereby promoting tumor cell invasion and migration 7. Therefore, we proposed that FKBP10 facilitated BC cells invasion and migration by downregulating lamin A. Indeed, wound healing and transwell assays showed that lamin A knockdown partially blocked the reduced migration and invasion induced by FKBP10 knockdown **(Fig. 6B-D).** Even in the Transwell assay with smaller-pore (3 and 5 μm) chambers, the downregulation of lamin A could partially restore the impaired migration ability of BC cells resulted from FKBP10 knockdown** (Fig. 6D).**

### FKBP10 is negatively correlated with lamin A expression in human BC

**Figure 7:**
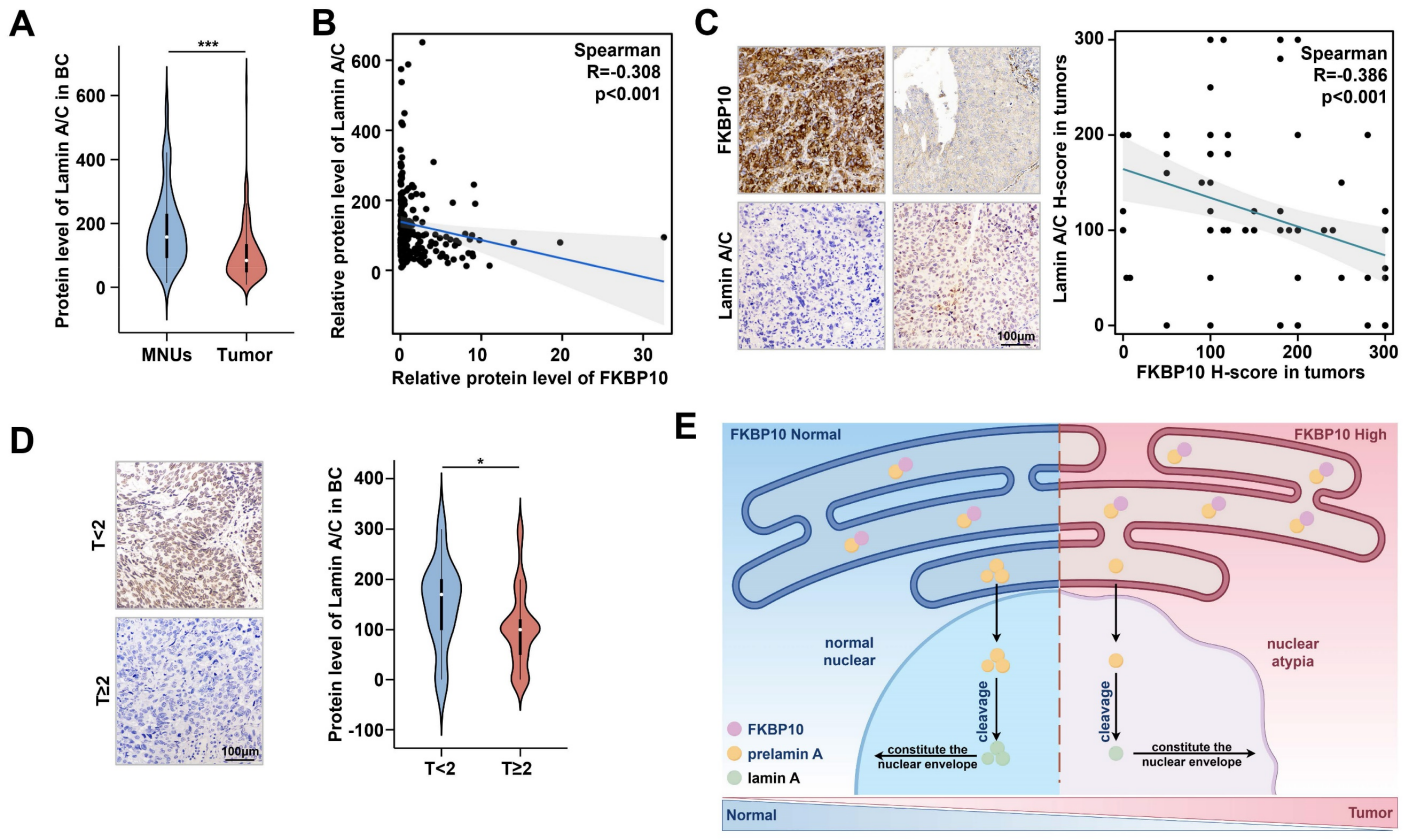
** The protein level of FKBP10 is negatively correlated with lamin A. A.** Expression of lamin A/C in MNUs and tumors from BC proteomic data in Chinese population.** B.** Spearman correlation analysis of FKBP10 and lamin A/C expression in tumors from bladder cancer proteomic data in Chinese population.** C.** Spearman correlation analysis of FKBP10 and lamin A/C expression in IHC score of BC tissue arrays.** D.** IHC results of lamin A/C in BC at different T stages. **E.** Schematic illustrating FKBP10's role in promoting BC progression by hindering prelamin A entry into the nucleus, thereby reducing lamin A expression.

To evaluate the clinical relevance of FKBP10 and lamin A in BC, we analyzed the proteomic data from 145 patients 23. We found that lamin A levels expression was significantly lower in BC tissues compared to that in adjacent normal tissues **(Fig. 7A)**, and FKBP10 expression was negatively correlated with lamin A level **(Fig. 7B)**. Consistent with these findings, IHC staining results of another 100 BC samples revealed an inverse relationship between FKBP10 and lamin A expression **(Fig. 7C)**. Moreover, lamin A expression was much lower in MIBC than that in NMIBC** (Fig. 7D),** which was consistent with the expression trends *in vitro* **(Fig. 5A)**.

## Discussion

Here, we revealed that the FKBP10/prelamin A/lamin A axis contributed to the nuclear atypia in BC, as well as BC progression. In BC, FKBP10 overexpression interacted with more prelamin A, then sequestered them in ER. Consequently, less prelamin A translocated into the nucleus and resulted in nuclear atypia, which enhanced the migration and invasive properties of BC cells **(Fig. 7E)**.

Nuclear atypia is a prominent feature of cancer cells and has been used as a diagnostic criterion for various cancers since the mid-18th century 25. It is widely recognized as a histopathological indicator associated with a higher recurrence risk and reduced patient survival. In BC, nuclear atypia serves as the standard for grading high-grade BC and is strongly linked to muscle invasion 4 and NMIBC recurrence 26. BC cells that infiltrate deeper layers of the bladder exhibit heightened nuclear polymorphism 27. While most studies have focused on the clinical correlations of nuclear atypia with BC progression and recurrence 28, 29, its underlying mechanism remains unclear. In this study, we found that FKBP10 could directly bind to prelamin A and inhibit its nuclear translocation. This led to the downregulation of lamin A, which in turn resulted in nuclear atypia, and promoted BC progression.

Lamin A, a key component of the nuclear lamina, is involved in maintaining nuclear structure and function 30. Its downregulation has been reported in various cancers and is associated with increased nuclear deformability and instability, which contributes to nuclear atypia 10, 11, 31. Previous studies have attributed lamin A loss to transcriptional regulation via the AKT pathway 32, ATR-dependent phosphorylation and degradation 33, and histamine-mediated signaling 34. In BC, lamin A expression has been reported to be lower in tumor tissues compared to normal bladder tissues, especially in NMIBC 35. However, the mechanisms underlying this downregulation have not yet been elucidated. Consistent with prior studies, our findings confirm lamin A downregulation in BC and uncover a novel mechanism: the decreased nuclear translocation of prelamin A, which results from FKBP10 overexpression.

Prelamin A is synthesized in the cytoplasm and transported into the nucleus via the nuclear pore complex 36. After entering into the nucleus, prelamin A undergoes farnesylation and is cleaved by ZMPSTE24 to form mature lamin A 6. In this study, we found that FKBP10 directly binds to prelamin A, inhibiting its nuclear translocation and subsequent cleavage into lamin A. Unlike previous studies that focused on structural abnormalities in prelamin A 37 or defects in the enzymatic activity of ZMPSTE24 31, 38, 39, our study highlights the importance of protein-protein interactions in regulating lamin A levels. This finding raises important questions about whether FKBP10 binding alters the structure of prelamin A and hampers its translocation out of the ER. Addressing these questions in future studies will help to further elucidate this regulatory mechanism.

Previous studies have reported that FKBP10 interacts with type I collagen to facilitate collagen maturation or binds to lactate dehydrogenase A (LDHA) to enhance the Warburg effect in renal cell carcinoma 21. In this study, we identified a new downstream effector of FKBP10. FKBP10 directly interacts with prelamin A, highlighting a novel role of FKBP10 in nuclear structure regulation. The function of FKBP10 in tumor progression varies by cancer type. For instance, FKBP10 has been reported to enhance stemness and invasion in lung cancer 20, promote glioma proliferation via the AKT-CREB-PCNA pathway 19, and drive colorectal cancer progression through transcriptional activation by circREEP3 22. In BC, we found that FKBP10 overexpression correlates with poor prognosis. FKBP10 knockdown significantly reduced invasion and migration *in vitro*, without affecting cell proliferation. Notably, FKBP10 knockdown led to a decrease in nuclear atypia. In an orthotopic bladder tumor model, FKBP10 knockdown significantly inhibited muscle infiltration and lung metastasis. These findings empahsize the pivotal role of FKBP10 in BC progression, and provide a strong basis for targeting FKBP10 as a potential therapeutic strategy in BC.

Targeting FKBP10 may have therapeutic potential, because the FKBP family exhibits rapamycin-binding ability 40. For example, FDA-approved rapamycin analogs such as everolimus, temsirolimus, and tacrolimus, which target FKBP12, have shown the antitumor efficacy in various cancers, including breast cancer, lung cancer and BC 41-43. However, toxicity remains a limitation of these drugs. Notably, studies have found that rapamycin only inhibits the first domain of FKBP10 13, which interacts with prelamin A. This specificity suggests a promising avenue for developing targeted therapies that restore prelamin A nuclear import while minimizing off-target effects. Future research should prioritize designing next-generation rapamycin analogs with improved safety profiles to exploit FKBP10 as a therapeutic target in BC.

## Conclusion

Our study highlights the significant role of the FKBP10/prelamin A/lamin A axis in regulating nuclear atypia, facilitating muscle invasion in BC, thus revealing it as a promising therapeutic target for BC.

## Supplementary Material

Supplementary figures and table.

## Abbreviations

BC: Bladder Cancer
MIBC: Muscle-Invasive Bladder Cancer
NMIBC: Non-Muscle-Invasive Bladder Cancer
FKBP10: FK506 Binding Protein 10
aa: amino acids
IHC: Immunohistochemistry
IP: Immunoprecipitation
MS: Mass Spectrometry
Co-IP: Co-Immunoprecipitation
IF: Immunofluorescence
PPIase: Peptidyl-Prolyl cis-trans Isomerase
ER: endoplasmic reticulum
