# Supplementary material for: FKBP10 Promotes the Muscle Invasion of Bladder Cancer via Lamin A Dysregulation
Source: Int J Biol Sci. 2025 Jan 1;21(2):758–71. doi: 10.7150/ijbs.105265 (PMC11705641; doi:10.7150/ijbs.105265)
Supplement: Supplementary file 1 — Supplementary figures and table. [file ijbsv21p0758s1.pdf]

Supplementary Materials for

FKBP10 promotes the muscle invasion of bladder cancer via prelamin A dysregulation

Xupeng Zhao *et al.*

*Corresponding author. Email: Hongzhao Li, urolancet@126.com; Xiubin Li, klootair@163.com; Xu Zhang, xzhang301@163.com

This PDF file includes:

Fig. S1 to S4
Tables S1

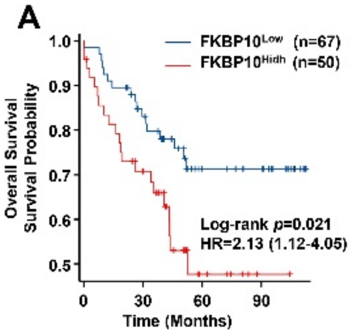

Fig. S1. FKBP10 is associated with poor prognosis in BC patients.
A. Kaplan-Meier curves showing the OS of patients with high or low expression of FKBP10 in the zhongshan cohort.

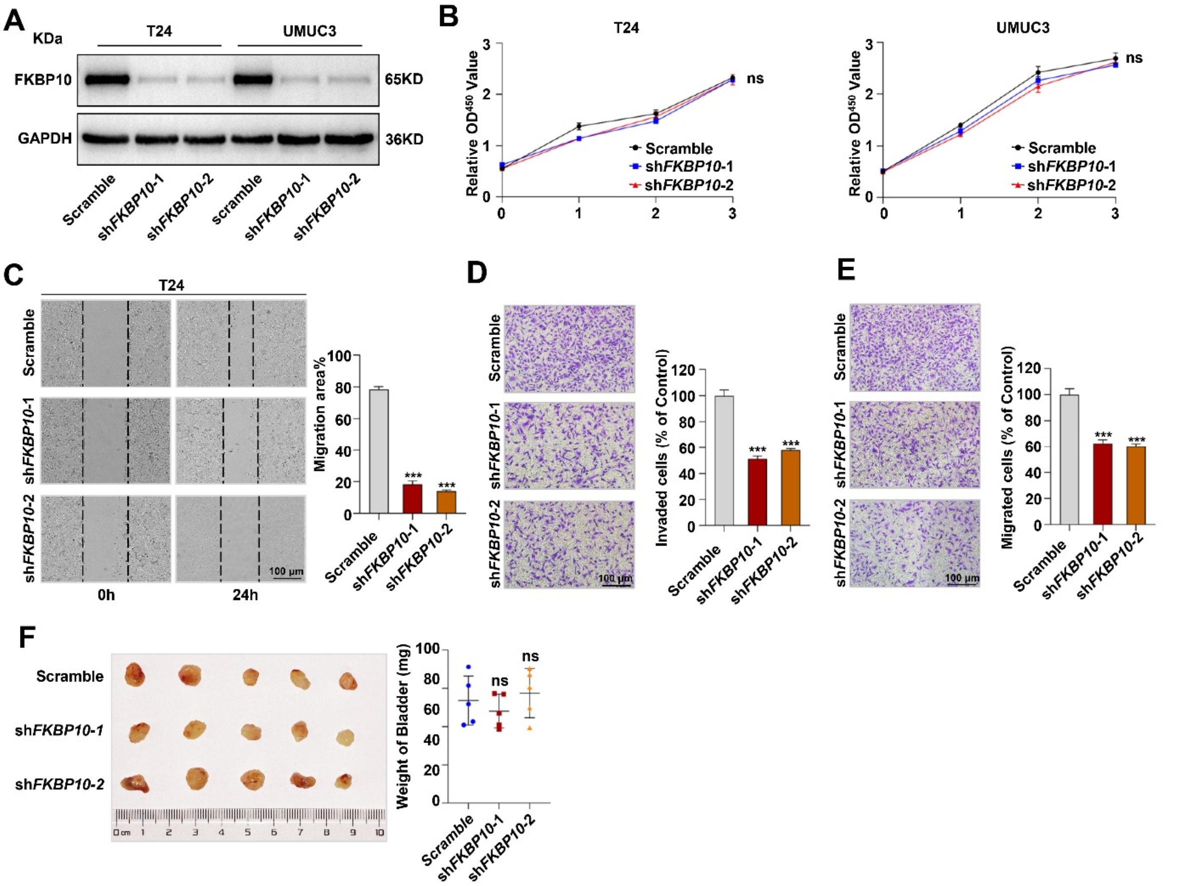

Fig. S2. FKBP10 influences the invasion and migration abilities of T24 cells.

A. Western blot to verify the knockdown efficiency of FKBP10 in T24 and UMUC3 cells. **B.** CCK8 experiment to verify the effect of knocking down FKBP10 on the proliferation ability of T24 and UMUC3 cells (n=3 per group). **C.** Representative pictures (left) and statistical results (right) of the wound healing assays of scramble or shFKBP10 T24 cells (n=3 per group). **D, E.** Representative pictures (left) and statistical results (right) of cell invasion and migration in Transwell experiments of scramble or shFKBP10 T24 cells (n=3 per group). **F.** Gross view of the subcutaneous xenograft tumor model of nude mice (left) and statistical analysis of tumor weight (right) (n=5 per group). Data are expressed as mean ± standard deviation. Statistical analysis was performed using unpaired two-tailed Student's t test. * p<0.05, ** p<0.01, *** p<0.001.

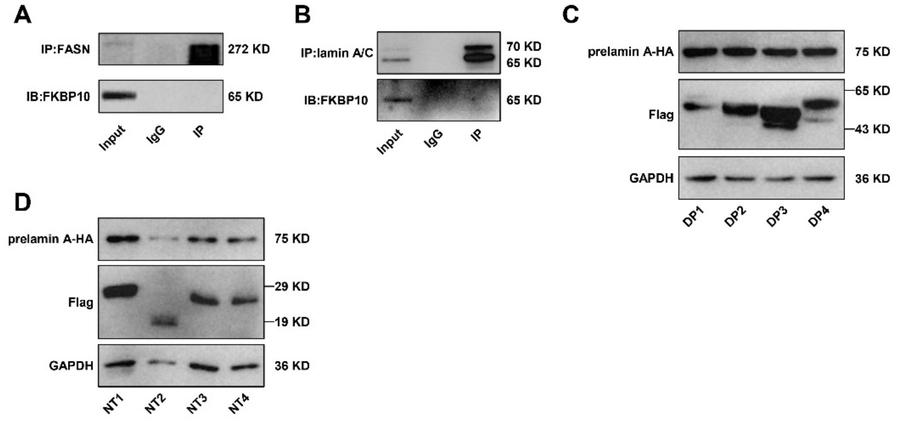

Fig. S3. FKBP10 does not interact with FASN and lamin A/C

A. Co-IP analysis of endogenous FKBP10 and FASN in UMUC3 cells. **B.** Co-IP analysis of endogenous FKBP10 and lamin A/C in UMUC3 cells. **C, D.** Input of truncated-FKBP10 and prelamin A Co-IP experiments in HEK293 cells.

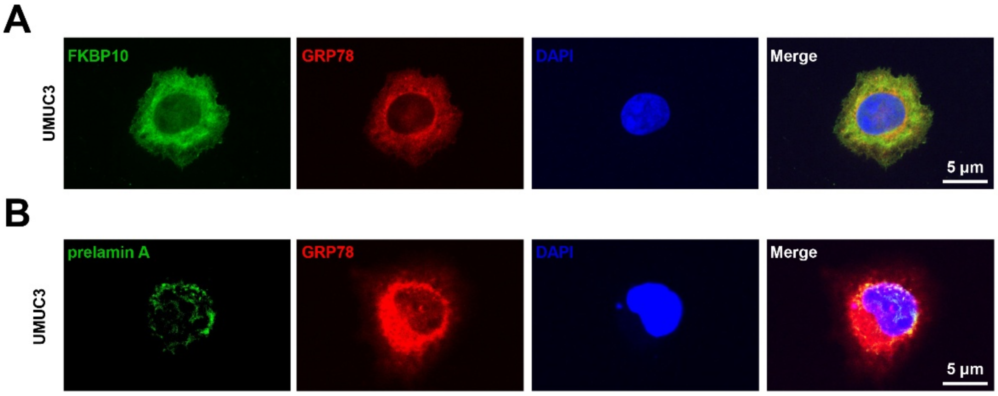

Fig. S4. FKBP10 and prelamin A are localized to the ER.
A. IF assays showed that FKBP10 located mainly in the ER in UMUC3 cells. FKBP10 was shown in green, while ER was marked by GRP78 and visualized in red. **B.** IF assays showed that prelamin A located in the endoplasmic reticulum and nuclear in UMUC3 cells. Prelamin A was shown in green, with the ER marked by GRP78 and visualized in red.

Table S1. Primers and oligonuceotides sequences.

**Interfering oligonucleotides**	5'→3'
sh*FKBP10*-1	CCACACCTACAATACCTATAT
sh*FKBP10*-2	CTACCACTACAACGGCACTTT
**Small interfering RNAs**	5'→3'
LMNA	
Forward primer	GCUGCGCAACAAGUCCAAUTT
Reverse primer	AUUGGACUUGUUGCGCACCTT
